# Aerobic exercise training combined or not with okra consumption as a strategy to prevent kidney changes caused by metabolic syndrome in Zucker rats

**DOI:** 10.1371/journal.pone.0269418

**Published:** 2022-06-03

**Authors:** Monique Marques da Silva, Moisés Felipe Pereira Gomes, Elizabeth de Orleans Carvalho de Moura, Mariana Matera Veras, Melina Chiemi Kubota, Ana Paula Takano, Ana Carolina Cardoso dos Santos, Carolina Gonçalves dos Reis José, Graziele Aparecida da Silva Souza, Naiara Magalhães Cardoso, Debora Estadella, Rafael Herling Lambertucci, Alessandra Medeiros

**Affiliations:** 1 Interdisciplinary Graduate Program in Health Sciences, Federal University of São Paulo, Santos, Brazil; 2 Laboratory of Environmental Air Pollution, Department of Pathology, University of São Paulo - School of Medicine, São Paulo, Brazil; 3 Biosciences Department, Federal University of São Paulo, Santos, Brazil; University Medical Center Utrecht, NETHERLANDS

## Abstract

The complications of Metabolic Syndrome (MetS) include kidney disease, and most dialysis patients are diagnosed with MetS. The benefit of exercise training (ET) for MetS treatment is already well defined in the literature, but the antidiabetic and antihyperlipidemic benefits of okra (O) have been discovered only recently. The aim of this study was to evaluate the effects of O and/or ET supplementation on renal function and histology; serum urea and creatinine value; inflammation (IL-6, IL-10, TNF-α) and oxidative stress in renal tissue. For this, 32 Zucker rats (fa/fa) were randomly separated into four groups of 8 animals each: Metabolic Syndrome (MetS), MetS + Okra (MetS + O), MetS + Exercise Training (MetS + ET), and MetS + Exercise Training and Okra (MetS + ET + O), and 8 Zucker lean (fa/+) rats comprised the Control group (CTL). Okra was administered by orogastric gavage 2x/day (morning and night, 100 mg/kg) and ET performed on the treadmill, at moderate intensity, 1h/day, 5x/week for 6 weeks. Although the renal function was not altered, the animals with MetS showed greater fibrotic deposition accompanied by a worse stage of renal injury, in addition to increased kidney weight. Although all interventions were beneficial in reducing fibrosis, only ET combined with O was able to improve the degree of renal tissue impairment. ET improved the anti-inflammatory status and reduced nitrite levels, but the combination of ET and O was more beneficial as regards catalase activity. Okra consumption alone did not promote changes in inflammatory cytokines and oxidative stress in the kidney. In conclusion, ET combined or not with O seems to be beneficial in preventing the progression of renal disease when renal function is not yet altered.

## Introduction

The metabolic syndrome (MetS) is characterized by a set of risk factors for cardiovascular disease, and central fat accumulation, high blood pressure, hyperglycemia, dyslipidemia, and insulin resistance (IR) are its main markers [1, 2].

The prevalence of MetS in Brazilian individuals reaches 35.5% [3]. These values are close to those found in the world, showing a direct relationship with sedentarism and eating habits, factors that increase the predisposition to cardiovascular manifestations [3, 4].

Among the various complications that are found in individuals with MetS, we can highlight chronic kidney disease (CKD), which is a major public health problem [5]. The pathophysiological mechanisms of CKD caused by MetS are not yet fully understood and it is not known whether the dysfunction is due to MetS or only to some components that lead to MetS [6, 7].

In general, the associated MetS factors lead to renal hyperfiltration and hyperperfusion, focal segmental glomerulosclerosis, glomerulomegaly, glomerular and tubule remodeling, reduced glomerular filtration rate (GFR), glomerular ischemia and tubular atrophy, thus characterizing CKD [6]. In addition to all these histological changes, renal oxidative stress is also observed [8]. An experimental study demonstrated an increase in the expression of cytokines tumor necrosis factor alpha (TNF-α), interleukin-6 (IL-6) and interleukin-12 (IL-12) *in vitro* investigations of renal cells in diabetic animals [9].

Measures to control CKD include prevention strategies and lifestyle changes based on the adoption of healthy eating habits and regular physical exercise [2, 10]. It is known that regular physical exercise has several positive effects on MetS factors, promoting an improvement in lipid metabolism, glucose and insulin sensitivity, as well as reducing blood pressure and decreasing the amount of adipose tissue [10]. In addition, aerobic exercise training performed by obese Zucker rats reduced renal oxidative stress [11], suggesting that physical exercise can help not only in MetS factors, but also in renal changes.

On the other hand, it is known that changes in eating habits are also able to assist in the treatment and prevention of MetS [2, 10]. Recently, the chemical properties of okra (*Abelmoschus esculentus L*. *Moench*) have been investigated, and in 2011 [12] the antidiabetic and antihyperlipidemic potential of this flavonoid-rich food were first described in diabetic rats.

Previous studies have shown that okra (O) consumption for 8 weeks improved renal function, reduced hyperfiltration, reversed renal fibrosis and improved the antioxidant effect [13]. These data suggest that both ET and O can be considered non-drug strategies for MetS treatment. However, studies about the effects of these therapies, applied alone or jointly, on structural and renal changes observed in MetS are still scarce in the literature.

Therefore, this study was designed to evaluate the effects of O supplementation combined or not with ET on inflammation, oxidative stress, renal function, and histology of animals with MetS.

## Materials and methods

For execution of the present study, all ethical principles of animal experimentation were applied, according to the National Council for Animal Experimentation Control (CONCEA), respecting the Brazilian legislation on animal experimentation (Federal Law # 1,794–2008). This project was submitted to the research ethics committee of the Federal University of São Paulo (UNIFESP), filed under CEUA # 3638241117.

### Animals

Eight lean male Zucker rats (fa/+) comprised the control group (CTL) and 32 obese male Zucker homozygous rats (fa/fa) were randomly separated into four experimental groups of 8 animals each: Metabolic Syndrome (MetS), Okra (MetS + O), MetS + Exercise Training (MetS + ET) and MetS + Exercise Training and Okra (MetS + ET + O). All rats were 10 weeks old and came from the Center for the Development of Experimental Models for Medicine and Biology (CEDEME) at UNIFESP. The animals were kept in cages containing three animals each, under controlled room temperature between 22 ± 2 ºC, inverted light/dark cycle of 12:12 hours, and fed a standard diet for laboratory rodents (Nuvilab CR-1) and water *ad libitum*.

### Okra preparation and administration

The okra pods (100 kg of Abelmoschus esculentus L. Moench.) were purchased in the municipality of Piacatu-SP, with the group of horticulturists Transvendrame (CNPJ 08.022.945 / 0001–16). Lat: -20.9984 Lon: -50.2983. The plant material was transported to Itupeva, all the dried okra pods were washed under running water, crushed and lyophilized by the company LioFoods. An okra from the aforementioned farm was used to make an exsiccata, which was made by employees of the Botany Department of the University of São Paulo and deposited in the USP herbarium with registration 22785.

Samples of okra (*Abelmoschus esculentus L*. *Moench*) were sprayed and lyophilized to be administered by orogastric gavage. The animals received a concentration of 100 mg/kg diluted in 1 ml of filtered water, 2 times a day (morning and night), 5 times a week, for the 6 weeks of the experimental protocol. The groups that did not receive okra were gavaged with 1 ml of filtered water so that all animals were subjected to the same conditions.

### Aerobic exercise training protocol

After a week of adaptation to the treadmill, an exercise tolerance test was performed, which consisted of a staggered protocol with an initial speed of 3 m/min for 5 minutes for warm-up and increments in the treadmill speed of 3m/min each 3 minutes, until the animal was exhausted [14–17]. The ET protocol lasted 6 weeks and was performed 5 days/week, 60 minutes/day, with an intensity of 70% of the speed obtained in the maximum effort test (moderate intensity) [18]. Halfway through the protocol (3 weeks), a new stress test was performed, only in groups submitted to exercise training to adapt the intensity of physical training. However, to assess the effect of interventions on tolerance to exertion, all animals were subjected to this test at the beginning and at the end of the intervention period.

### Evaluation of blood pressure, glycemic, lipid and body mass profiles

The profiles were identified for 7 weeks (before and during the 6 weeks of intervention), one measurement per week, on the same day and time for all animals in the study.

The measurement of animals’ caudal blood pressure was performed using the BP-2000 series II^™^ Blood Pressure Analysis System, in the first hours of the light cycle and before the animals were gavaged with water or O and ET, to avoid stress-related pressure variations

Blood glucose, triglycerides and total cholesterol were measured by collecting blood from the animal’s tail and using specific reagent strips of the Accutrend^®^ Plus device. HDL cholesterol was quantified using the Labtest kit and following the manufacturer’s instructions. For LDL calculation we used the Friedewald method.

The animals’ body mass was measured weekly on a Gehaka^®^ BK5002 class II scale.

#### Insulin Tolerance Test (ipITT)

The animals remained fasting for 6 hours to perform the ipITT. After collecting blood from the tail, 2 IU/Kg (ip) of insulin was administered and blood glucose was again assessed at 5, 10, 15, 20 and 30 min.

#### Glucose Tolerance Test (ipGTT)

After 8-hour fast, blood glucose was analyzed at time 0 and glucose 2.0 g/kg (ip) was administered. Additional blood samples were collected at 30, 60, 90 and 120 minutes for blood glucose analysis.

### Euthanasia

At the end of the experimental period, the rats were kept without manipulation for 24 hours, were anesthetized with thiopental (30mg/kg, ip) and lidocaine (5 mg/kg, ip) and euthanized by decapitation.

During euthanasia, blood was collected and centrifuged in an Eppendorf^®^ Centrifuge 5702^®^ for 20 minutes at a speed of 20000 rpm. The serum was stored at -80 ºC for biochemical analysis of urea and creatinine to be performed later.

The kidneys were also dissected out and weighed and the right kidney was stored at -80 ºC for further biochemical analysis. The left kidney was stored in 4% buffered formaldehyde for 24 hours and then transferred to 70% alcohol, where it remained until the beginning of histological processing.

After removing all the tissues involved in this experiment, the animals’ right tibia was removed, and its length measured with the aid of a caliper for later normalization of the previously mentioned tissue weights.

### Renal function analysis

For this evaluation, the parameters of urea and plasma creatinine measurement were used using commercial kits Labtest Diagnostica^®^. In addition, 24-hour diuresis was measured using the metabolic cage.

### Renal histopathological analysis

The left kidney was preserved in 4% formaldehyde for a period of 24 hours, after which it was immersed in 70% dehydrated alcohol and fixed in paraffin. Sections of 5 μm were obtained to compose the slides, which were stained with Periodic Acid-Schiff (PAS) and picrosirius red for identification of renal structures.

The qualitative histopathological evaluation was conducted according to the *Society of Toxicologic Pathology* [19] recommendations. The following parameters were blindly assessed by two observers according to the degrees of intensity in the fields analyzed: change in the proportion of the cortical and pelvic regions; morphological changes in the glomerular unit and renal tubules; cell degeneration or death in the following compartments: glomerulus, renal tubules.

The slides were analyzed by optical microscopy and classified according to kidney damage [20], where 0 means kidney damage less than 5%; I, kidney damage between 5 and 25%; II, kidney damage between 25 and 50%; III, kidney damage between 50 and 75%; IV, kidney damage greater than 75%.

Quantitative analysis of renal fibrosis was performed on slides stained with Picro-Sirius Red and of the glomerular area on HE-stained slides. First, the slides were digitized using the digital slide scanner Pannoramic SCAN (3DHistech Ltd., Budapest, Hungary), later visualized in the Software Pannoramic Viewer version 1.15.4, which allowed to capture images that were quantified in the Image J program (1.52ª). The results of renal cortex fibrosis were obtained by averaging 15 photographs per slide, from each group, by counting points, using 20x magnification. The results of the glomerular area, which involves the glomerular capsule area, were obtained from 3 photos per slide stained with PAS, at a 20x magnification.

### Oxidative stress analysis

Prior to the oxidative stress analysis, proteins from kidney samples were measured as described by Bradford in 1976 [21].

#### Total Antioxidant State analysis

The Total Antioxidant State (TAS) was determined using the method proposed by Erel in 2004. In this method, the antioxidant power of the samples is evaluated against potent reactions of free radicals, which are initiated by the production of the hydroxyl radical. The reading was performed with a spectrophotometer (Biotek, Elx800) at 440 nm. The results are expressed as μmol Trolox Eq/L [22].

#### Total Oxidizing State analysis

The Total Oxidizing State (TOS) was determined using the method proposed by Erel in 2005. The samples were homogenized. The color intensity of the reaction, mediated by the ferrous-ion complex with xylenol orange in an experimental acid medium, measured with a spectrophotometer (Biotek, Elx800) at 560 nm, is related to the total amount of oxidizing molecules present in the sample, which react with the ferrous ion-o-dianisidine complex. The oxidation reaction is catalyzed by the glycerol molecules present in the medium. The test is calibrated with hydrogen peroxide (H_**2**_O_**2**_) and the results are expressed as μmol H_**2**_O_**2**_ Eq/L [23].

#### Total superoxide dismutase enzyme activity determination

The total SOD activity was monitored by the reduction of cytochrome *c* (0.15 g/L) by the superoxide radicals generated in the xanthine-xanthine oxidase system. The measurement was performed at 550 nm and 37 °C, according to the protocol of Flohe and Otting in 1984 [24]. First, the blank was determined, finding the appropriate amount of buffer and xanthine oxidase so that the absorbance was as close as possible to 0.025. That done, for each assay, 1 μl of sample, 195 μl of solution A (cytochrome C, EDTA and xanthine in phosphate buffer) and the amounts found in the sodium phosphate buffer (50 mM) and xanthine oxidase blank were added. The activity was expressed in U per mg of protein following the equation: [(absorbance of blank—absorbance of the sample) / (absorbance of blank / 1.4)] x dilutions / mg of protein. The reading was performed with a spectrophotometer (SpectramaxM2) at 406 nm.

#### Catalase enzyme activity determination

The catalase activity tests were performed according to the method described by Aebi in 1984 [25]. In this, enzyme activity was determined by the consumption of H_2_O_2_ at 230 nm and 25 °C. The reaction occurred at pH 7.4 and was monitored for 5 min. In each assay, 1 μl of sample, 15 μl of 50 mM KPO buffer, and 189 μl of 10 mM H_2_O_2_ were added. To calculate the activity, the following equation was used: (Δ absorbance / 0.071) x dilutions / mg of protein. The reading was performed with a spectrophotometer (SpectraMax M2) at 204 nm.

#### Glutathione peroxidase activity determination

The activity of the enzyme glutathione peroxidase was carried out by monitoring the consumption of NADPH at 340 nm (SpectraMax M2). A final concentration of 0.25 mM NaN_3_, 0.25 U / ml glutathione reductase, 1 mM GSH, 1 μl sample, 184 μl Stoch Buffer (143 mM sodium phosphate and 6.3 mM EDTA–pH 7.5) and 0.12 mM NADPH were added to each test. After incubation at 37 ºC for about 30 seconds, 5 μl of TBHP (t-butyl hydroperoxide) was added and monitored for 5 minutes. 200 μl of Stoch Buffer were used as a reference. The results were expressed in nmol of TBHP reduced per minute, per mg of protein, considering the NADPH extinction coefficient of 6.22 nM^-1^ cm^-1^ [26]. Thus, GPx activity was obtained according to the equation: GPx activity = (Δ absorbance / 6.22) x dilutions / mg of protein.

#### Lipid peroxidation determination

The thiobarbituric acid (TBARS) results in the concentration of malondialdehyde (MDA), a product of lipid peroxidation, of samples and which is a substance that reacts with thiobarbituric acid and produces specific staining for analysis in a spectrophotometer. 250 μl of thiobarbituric acid (1% in 50mM NaOH) and 250 μl of 25% HCL were added, and for the standard, 250 μl aliquots of 10^−1^ mM 1,1,3,3-tetramethoxypropane were added. The tubes containing the mixture were incubated in a boiling bath (100 °C) for 10 minutes and cooled in an ice bath. Then 730 μl of butanol were added to each tube and vortexed until there was a complete transfer of the pink color from the lower layer to the upper layer. Centrifugation was then performed for 5 minutes at 12,000 rpm. The butanol phase was removed, and the absorbance was evaluated at 532 nm in a spectrophotometer. The TBARS concentration was then determined using the MDA molar extinction coefficient (ε = 1.56 x 10^−5^ M^-1^.mL), using the TBARS concentration = (absorbance / 0.156) x dilution = nm / mg protein [27].

#### Assessment of nitrite content in kidney

Analysis of nitrite content in kidney of animals was conducted through the previously proposed method [28] in which nitrite reacts with sulfanilamide under acidic conditions and the reaction product reacts with N-chloridrate (l-naphtyl) ethylenediamine (NED) that generates a compound with an intense red color (Griess reagent). For each test, 100 μL of sample was added to the same volume of Griess test solution (containing 1% sulfanilamide, 0.1% of NED and 2.5% phosphoric acid (H_3_PO_4_) and then incubated for 10 min at 25°C. After the incubation period, the absorbance reading at 550 nm was measured, along with a sodium nitrite curve generated from concentrations of 100, 50, 25, 12.5, 6.25, 3.12, 1.56 and 0.78 μM. Calculations were performed taking into consideration the values obtained in the absorbances of the samples and compared to values obtained in sodium nitrite curve (values were presented as μM/mg protein). Absorbance readings were performed by a spectrophotometer (Molecular Devices—Espectra Max 190).

#### Cytokines quantification in renal tissue

To analyze the inflammatory profile in renal tissue, protein extraction was performed using the homogenizer (omni model TH-02). Homogenization was performed with 100 mg of tissue in 0.8 mL of specific buffer: 100mM Trizma base, 20mM EDTA, 100mM sodium fluoride, 100mM sodium pyrophosphate, 10mM sodium phenylmethylsulfonyl fluoride (PMSF) 2mM and 0.1 mg of aprotinin per ml. Following the addition of 8 mL of Triton-x 10% and maintained on ice for 30 minutes, centrifuged at 14,000 rpm at 4 ºC for 40 minutes. Supernatants were collected and the cytokines IL-6, IL-10 and TNF-α were assayed by the ELISA method, using Quantikine, R&D System kits (Minneapolis, MN, USA). And the proteins adjusted by Bradford, 1976 [21]. The samples were evaluated according to the manufacturer’s instructions.

## Statistical analysis

Microsoft Excel^®^ 365 Pro Plus software was used to tabulate the data. The data identified as outliers by the Grubbs test (0.05%) were removed, and the others submitted to normality evaluation by the Kolmogorov-Smirnov and Shapiro-Wilk tests. We used two-way ANOVA to characterize the sample and one-way ANOVA to analyze the other data. When differences between groups were detected, they were compared using a post-hoc Bonferroni test. In all the results presented, 5% was adopted as the limit of statistical significance (p <0.05) and the results were expressed as mean ± standard error of the mean (sem). Statistical analysis software Graphpad Prism^®^ 6.0 was used for the statistical analysis and graphs.

## Results

### MetS diagnostic parameters

**Fig 1:**
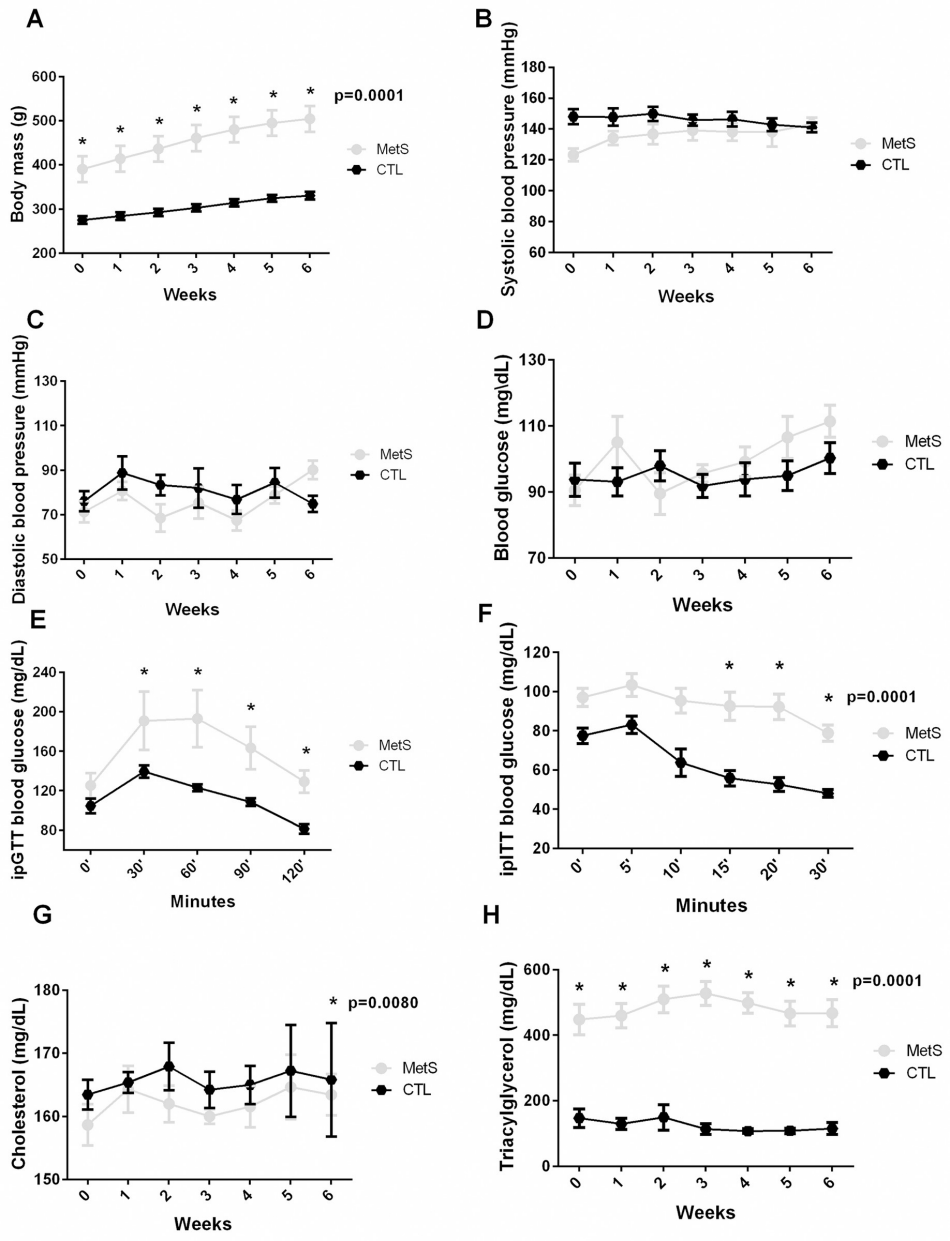
MetS diagnostic parameters—Control group. **(A)** body mass (g) (p = 0.0001), **(B)** systolic blood pressure (mmHg), **(C)** diastolic blood pressure (mmHg), **(D)** blood glucose (mg/dL), **(E)** ipGTT (minutes), **(F)** ipITT (minutes) (p = 0.0001), **(G)** cholesterol (mg/dL) (p = 0.0080), **(H)** Triacylglycerol (mg/dL) (p = 0.0001). *vs. CTL. Statistic: Two-Way ANOVA, p<0.05.

Animals with MetS, despite not showing changes in blood pressure (Fig 1B, p = 0.2371 and 1C, p = 0.1971) and blood glucose (Fig 1D, p = 0.2970), developed glucose intolerance (Fig 1E, p = 0.0001), insulin intolerance (Fig 1F, p = 0.0001), elevation of total cholesterol values (Fig 1G, p = 0.0080), increased triacylglycerol (Fig 1H, p = 0.0001) and increased body mass (Fig 1A, p = 0.0001), thus confirming the development of MetS.

**Fig 2:**
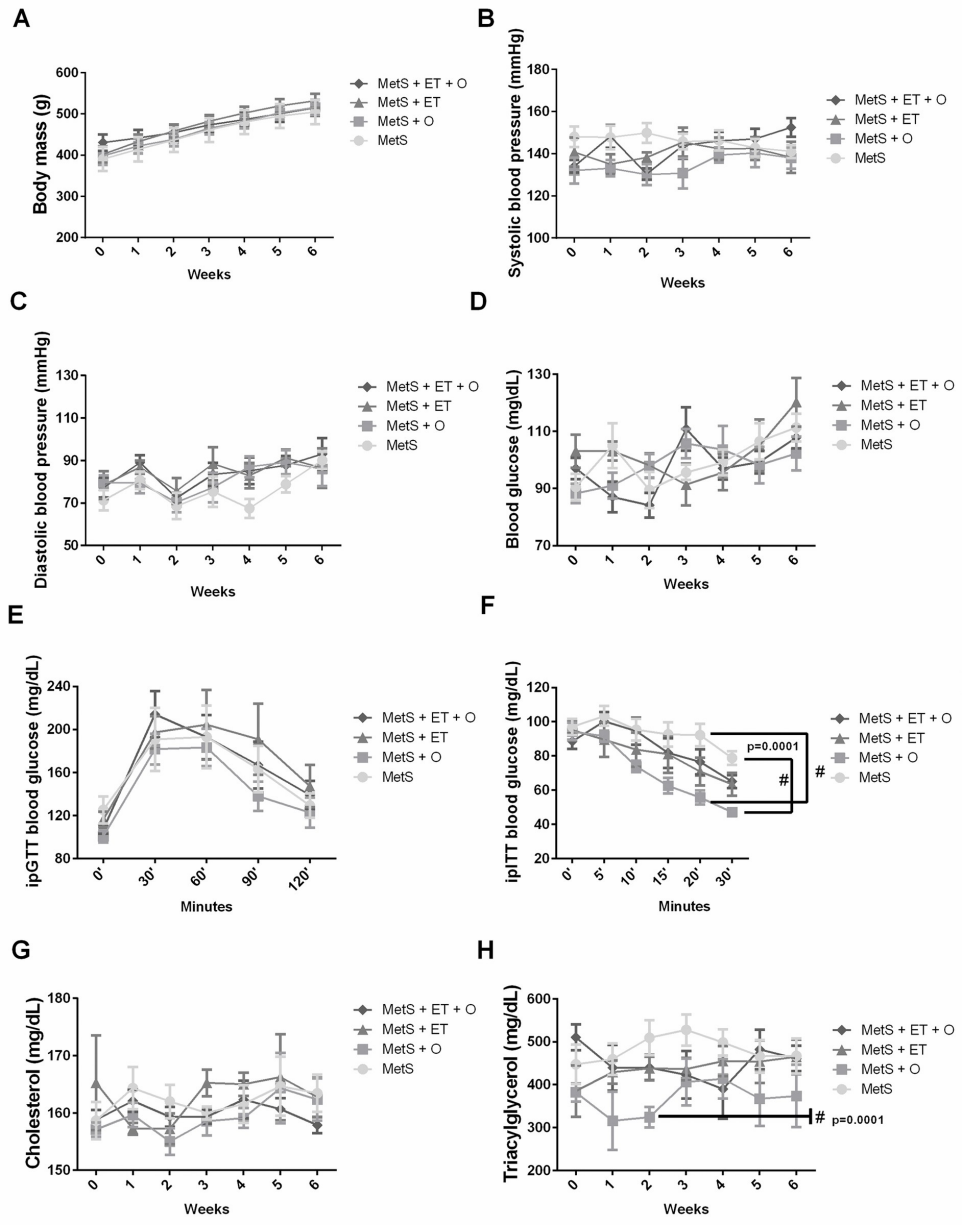
MetS diagnostic parameters—MetS group. **(A)** body mass (g), **(B)** systolic blood pressure (mmHg), **(C)** diastolic blood pressure (mmHg), **(D)** blood glucose (mg/dL), **(E)** ipGTT (minutes), **(F)** ipITT (minutes) (p = 0.0001), **(G)** cholesterol (mg/dL), **(H)** Triacylglycerol (mg/dL) (p = 0.0001). **#** vs. MetS. Statistic: Two-Way ANOVA, p<0.05.

The interventions did not promote changes in body mass (Fig 2A, p = 0.2291), blood pressure (Fig 2B and 2C, p = 0.9415), blood glucose (Fig 2D, p = 0.0720) and total cholesterol (Fig 2G, p = 0.9356). However, the MetS + O group showed improvement in insulin intolerance (Fig 2F, p = 0.0001), in addition to an improvement in triacylglycerol concentration (Fig 2H, p = 0.0001). MetS + ET and MetS + ET + O groups showed partial improvement in insulin intolerance, but it did not differ from the MetS (Fig 2F, p = 0.9945).

### Renal histology

**Fig 3:**
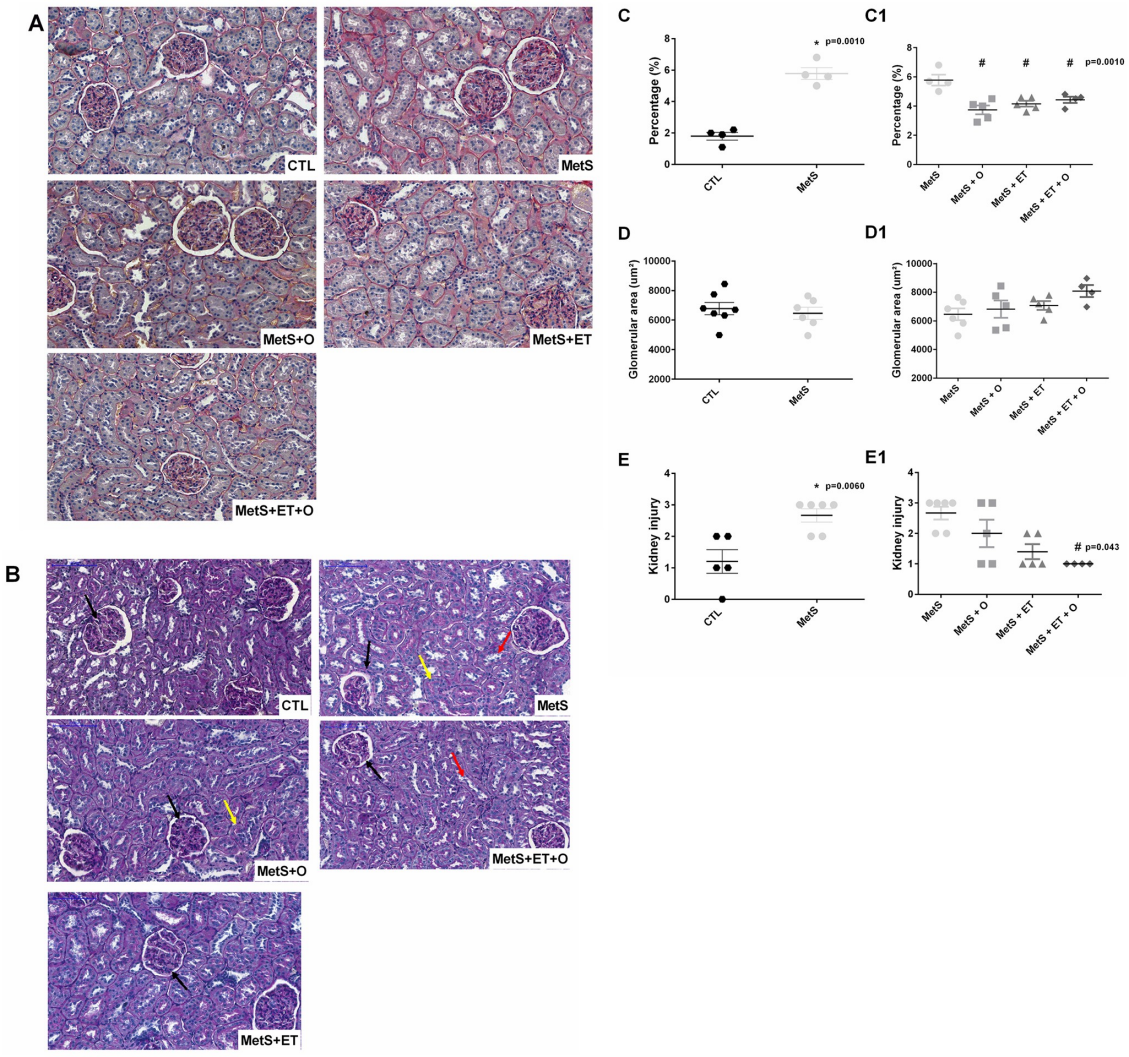
Renal histological analysis. **(A)** Photomicrographs representative of renal fibrosis, **(B)** Photomicrographs representative of other kidney injuries, **(C/C1)** Percentage of renal fibrosis (p = 0.0010 and p = 0.0010), **(D/D1)** Glomerular area, **(E/E1)** Degree of kidney injury (p = 0.0060 and p = 0.043). **Black arrow–**glomerular atrophy, **Yellow arrow–**tubular dilation, **Red arrow–**tubular degeneration. *vs. CTL; #vs. MetS. Statistic: One-Way ANOVA, p<0.05.

In renal histological sections, it was possible to observe that the MetS group had a higher percentage of fibrosis than the CTL group (p = 0.0001) (Fig 3A and 3B). The interventions had a partial effect on this parameter since the percentage of fibrosis was lower in the MetS + O, MetS + ET and MetS + ET + O (p = 0.0010) groups than in the MetS group (Fig 3C and 3C1).

Regarding the glomerular area, there were no differences between the groups (p = 0.5993) (Fig 3D and 3D1). However, when the degree of kidney damage was assessed, a greater degree was observed in the MetS group than in the CTL group (p = 0.0060), and only the group submitted to both interventions simultaneously (MetS + ET + O) showed improvement in this parameter (p = 0.043) (Fig 3E and 3E1).

### Renal function

**Fig 4:**
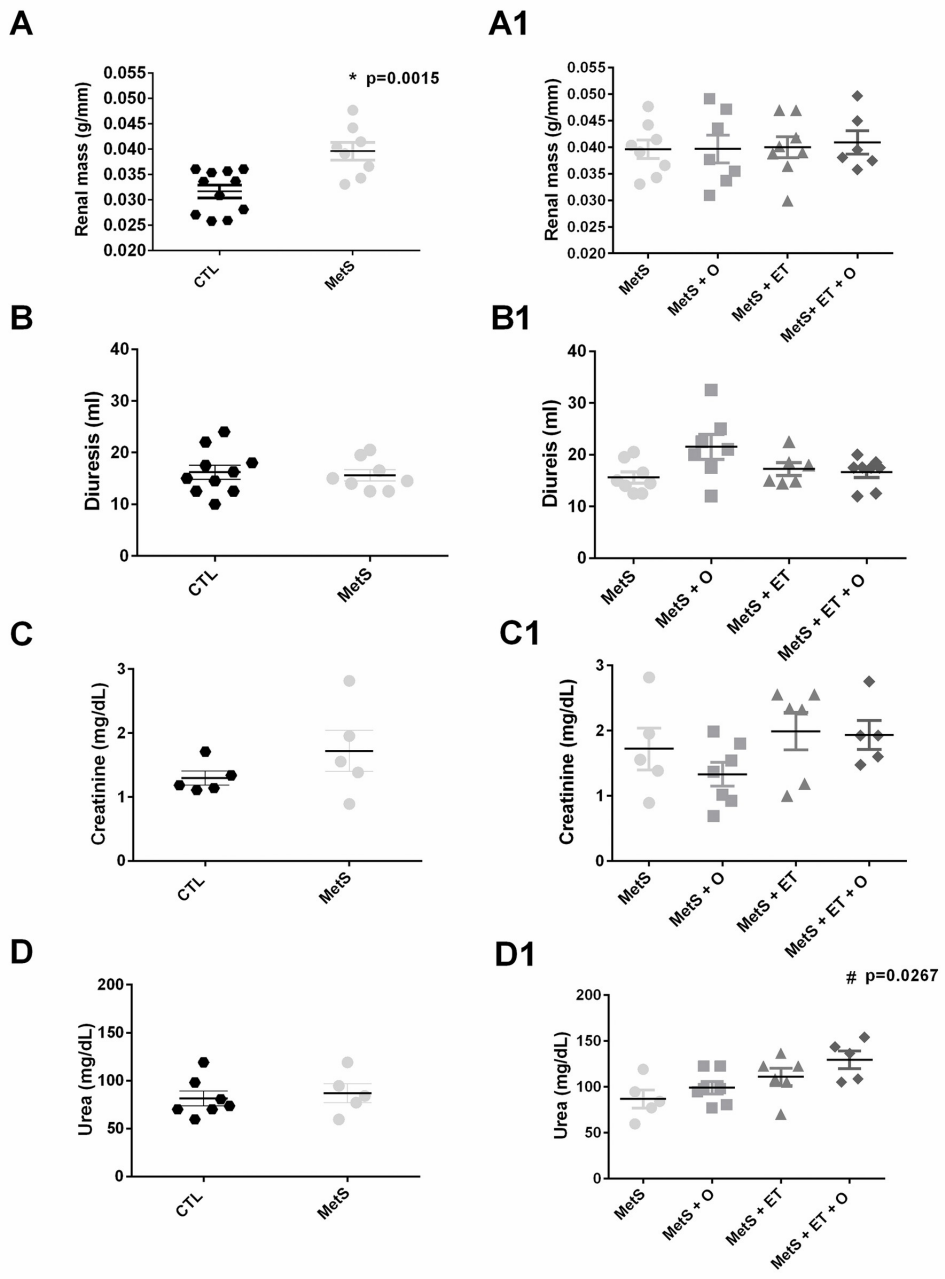
Analysis of renal function. **(A/A1)** Renal mass (p = 0.0015), **(B/B1)** Diuresis, **(C/C1)** Creatinine, **(D/D1)** Urea (p = 0.0267). #vs. MetS. Statistic: One-Way ANOVA, p<0.05.

The renal weight was greater in the MetS group than in the CTL group (p = 0.0015), and none of the interventions was able to change this parameter (p = 0.9743) (Fig 4A and 4A1). Regarding the volume of 24-hour diuresis presented by animals, no significant changes were observed across the groups (Fig 4B, p = 0.7476 and 4B1, p = 0.0514), neither in creatinine concentrations (Fig 4C, p = 0.2481 and 4C1, p = 0.2269). However, urea levels were higher in the MetS + ET + O group than SM group (Fig 4D and 4D1, p = 0.0267).

### Inflammatory cytokines in renal tissue

**Fig 5:**
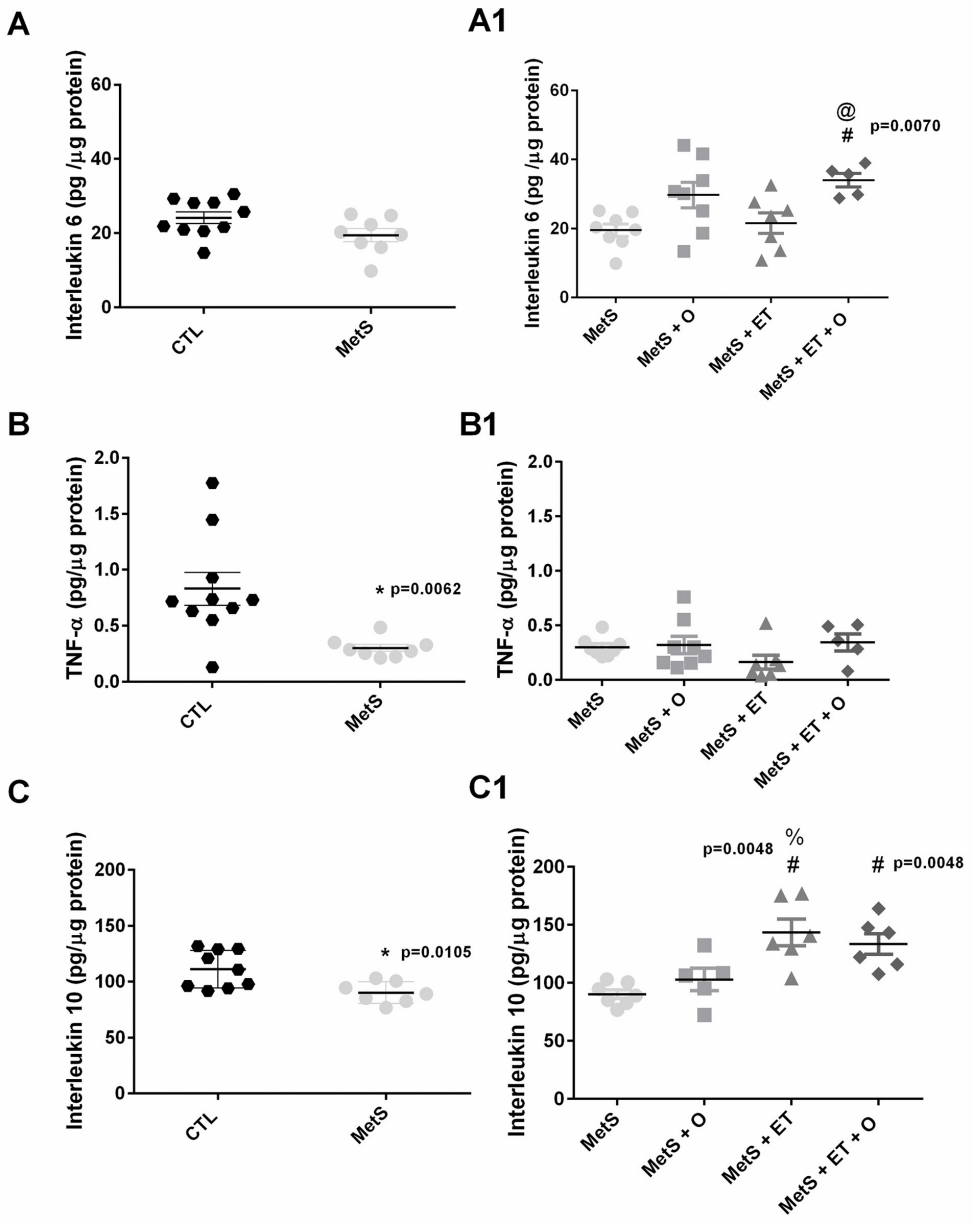
Analysis of inflammatory cytokines in renal tissue. **(A/A1)** Interleukin 6 (p = 0.0070), **(B/B1)** TNF-α (p = 0.0062), **(C/C1)** Interleukin 10 (p = 0.0048). *vs CTL, #vs. MetS, %vs. MetS + O, @vs. MetS + ET. Statistic: One-Way ANOVA, p<0.05.

Regarding IL-10 concentration, the MetS group differ from the CTL group (Fig 5C, p = 0.0105), and IL-10 concentration was higher in the MetS + ET group than both in the MetS and MetS + O groups (Fig 5C1, p = 0.0048). The MetS + ET + O group it was higher only compared to the MetS group (Fig 5C1, p = 0.0048).

When the TNF-α concentration in the renal tissue was evaluated, a lower concentration was observed in the MetS group than in the CTL group (Fig 5B, p = 0.0062), and the interventions were not able to change this concentration (Fig 5B1, p = 0.2334). Regarding IL-6 concentration, the MetS group did not differ from the CTL group (Fig 5A, p = 0.0649) and only the interventions used together (MetS + ET + O) were able to increase this concentration (Fig 5A1, p = 0.0070).

### Oxidative stress in renal tissue

**Fig 6:**
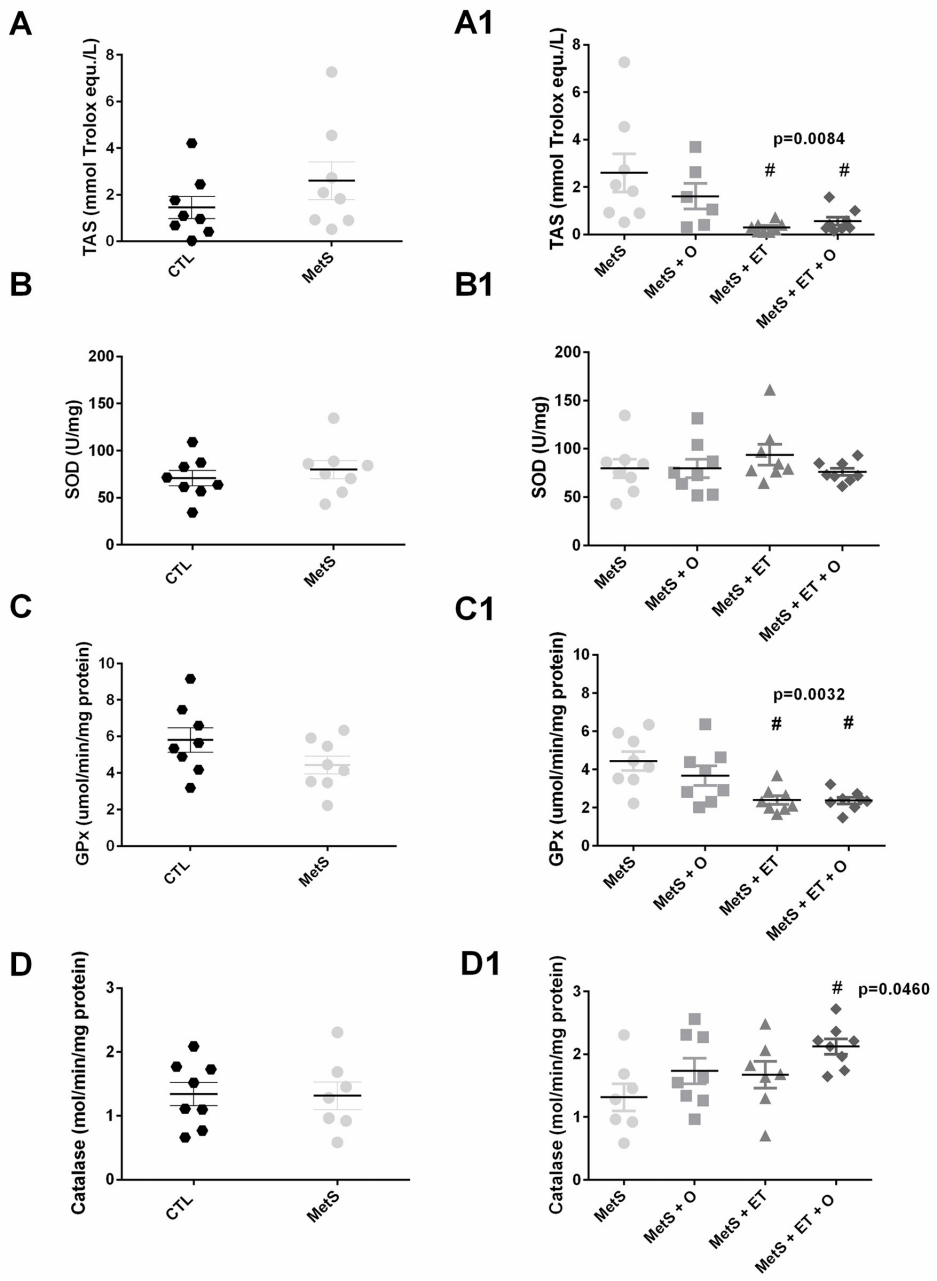
Analysis of antioxidative enzymes. **(A/A1)** TAS (p = 0.0084), **(B/B1)** SOD, **(C/C1)** GPx (p = 0.0032), **(D/D1)** Catalase (p = 0.0460). #vs. MetS. Statistic: One-Way ANOVA, p<0.05.

When TAS (Fig 6A, p = 0.2399), SOD (Fig 6B, p = 0.4850), GPx (Fig 6C, p = 0.1233) and catalase (Fig 6D, p = 0.9200) were analyzed, no differences were observed between the MetS and CTL groups. However, TAS (Fig 6A1, p = 0.0084) and GPx (Fig 6C1, p = 0.0032) were lower in the MetS + ET and MetS + ET + O groups than in the MetS group, while SOD (Fig 6B1, p = 0.5126) was not different across the groups. Catalase activity was increased only in the MetS + ET + O group as compared to the MetS group (Fig 6D1, p = 0.0460).

**Fig 7:**
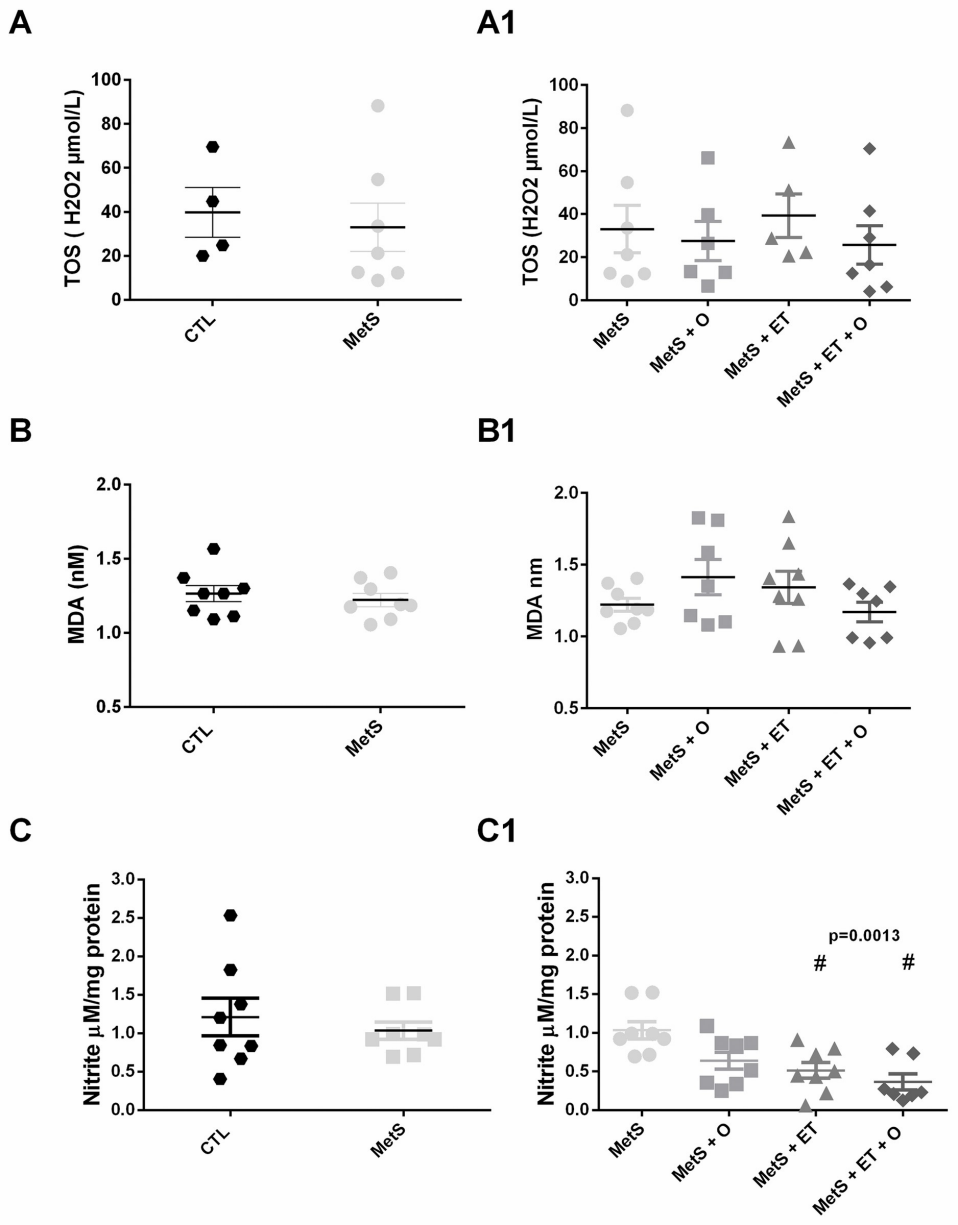
Analysis of oxidant enzymes. **(A/A1)** TOS, **(B/B1)** TBARS, **(C/C1)** Nitrite (p = 0.0013). #vs. MetS. Statistic: One-Way ANOVA, p<0.05.

There were also no differences across groups in TBARS (Fig 7B and 7B1, p = 0.2651) and TOS (Fig 7A and 7A1, p = 0.7948) analyses. In nitrite analysis, the MetS group did not show any difference as compared to the CTL group (Fig 7C, p = 0.5212). However, the ET groups were also different from the MetS group (Fig 7C1, p = 0.0013).

## Discussion

Our study aimed to investigate the changes caused in the kidney of animals with MetS and whether aerobic ET and O consumption were able to mitigate or prevent such changes. To do so, however, it was necessary to confirm whether the animals used would actually develop MetS in the first place. For the clinical diagnosis of MetS, three or more of the five factors described by the Adult Treatment Panel of the National Cholesterol Education Program (NCEP-ATPIII) are required, and we observed that our Zucker rats did develop IR, obesity and increased cholesterol and triglycerides. Therefore, it was possible to characterize them as animals with MetS.

Regarding the main findings of the present study in the animals’ kidneys, we can mention that MetS reduced TNF-α values in the renal tissue, promoted greater fibrotic deposition accompanied by a worse stage of renal injury, in addition to increasing kidney weight. As a result of the interventions, we observed that ET combined or not with O consumption increased IL- 10 concentration, reduced TAS, GPx and nitrite, and partially prevented the increase in the percentage of renal fibrosis. In addition, ET combined with O increased the urea concentration (but still within the normal range) and, in the kidneys, increased the IL-6 concentration and reduced catalase, in addition to preventing the increase in the degree of kidney damage. On the other hand, consumption of O alone was not able to modify cytokine concentrations and oxidative stress parameters in animals’ kidneys, but partially prevented the increase in the percentage of renal fibrosis.

Renal changes caused by MetS have already been described in the literature [6, 7, 29–32] and the causes seem to be multifactorial. However, it is known that IR and obesity promote endothelial dysfunction and activation of the renin angiotensin aldosterone system, causing kidney damage [32]. In our study, we observed that animals with MetS had higher kidney weight, accompanied by a higher percentage of fibrosis and a worse degree of renal damage, as previously observed in diabetic, obese and MetS animals [8, 33–36].

Other studies have already shown increased kidney weight in obese animals [33, 37], so we believe that obesity overload and renal fibrosis are responsible for this change. Renal tissue lesions, on the other hand, occur during two main pathological stages: the initial stage, which is hypertrophy, and sclerosis, which is the late stage. In the initial stage, glomerular cell proliferation occurs, which promotes hypertrophy. Glomerulosclerosis and tubulointerstitial fibrosis occur at a later stage [38]. In our study, we observed low levels of tubulointerstitial fibrosis, suggesting that these animals are in the early stage of damage. Another interesting finding is that the CTL group also presented renal atrophy, but with severity I, suggesting that it is normal for the animal.

In CKD, the structural alteration is accompanied by a reduction in renal function [5]. However, our data indicated that animals with MetS presented greater renal damage but no change in kidney function, which suggests that the damage, or the time of installation may not have been long enough to trigger such impairment. Muhammad et al. [11] also did not observe changes in the renal function of obese sedentary and trained Zucker rats. In a study with humans with CKD, renal function did not change either with the combination of aerobic exercise and caloric restriction [39].

Dyslipidemia is found in most patients with CKD, even in the early stages and is related to the decline in renal function [40, 41]. Previous data demonstrated that hyperlipidemia was slightly associated with renal pathology and that the increase in plasma lipids alone was not sufficient to cause significant renal damage [42]. It was also reported that the more severe the diabetes, the more severe the renal dysfunction [42]. In fact, hyperglycemia is associated with CKD development [43], which can promote changes in renal function [5]. IR also contributes to the progression of kidney disease due to the worsening of hemodynamics generated by the activation of the sympathetic nervous system, sodium retention and negative regulation of the natriuretic peptide system [44]. It is worth mentioning that our animals developed dyslipidemia, but not hyperglycemia, suggesting that IR associated with dyslipidemia may be the trigger for the injury found.

Recent data [45] have shown that okra intake for 8 weeks was beneficial in reducing plasma glucose, triglyceride levels and total cholesterol in diabetic individuals. No adverse side effects have been observed and may represent an adjunct therapy in the treatment of type 2 diabetes. Recent study [46] have shown that the consumption of hibiscus, a flower with flavonoid compounds and belonging to the okra family, can improve proteinuria and protect renal function by improving immune response, antioxidation, reducing inflammation, protecting renal tubular epithelial cells, and inhibiting renal fibrosis. In diabetic animals [13], subfractions of O improved nephropathy and reversed diabetic kidney fibrosis. Our results showed that all interventions were effective in improving fibrosis in renal tissue and the intervention of O associated with exercise was able to reduce the severity of renal injury in these animals. These data reaffirm the benefits of O in injuries caused by MetS.

Obesity, combined or not with MetS, creates a higher risk of developing CKD, predisposing to a pro-inflammatory state, increased IL-6 and TNF-α and possible progression of kidney injury to terminal CKD [47, 48]. A previous study showed that the levels of these cytokines were increased in the renal tissue of animals with MetS [38], a result not found in our study. Data show that nitrite was elevated in the renal tissue of diabetic animals [49] and when induced to inflamed [50] when compared to control animals. However, previous study [51] observed serum TNF-α levels were high in patients with type 2 DM, which did not occur in glucose-intolerant patients suggesting that TNF-α is not or is less affected in these individuals. In our study, we did not find high levels of TNF-α in the kidney of animals with MetS and we can suggest that this did not occur because they were not diabetic.

Therefore, the change in lifestyle seems to be a great alternative to reduce the harmful effects caused by MetS. ET brings several benefits through anti-inflammatory and antioxidant actions. Its antioxidant effect is due to the suppression of inflammatory pathways, which inhibit the sources of generation of reactive oxygen species. Exercise also generates activation of transcription factors sensitive to reduction-oxidation reactions (REDOX) improving the expression and activity of SOD, GPx and catalase [52]. In addition, ET releases IL-6 through muscle contraction, increases IL-10 levels and decreases circulating levels of TNF-α [53]. Evidence shows that aerobic exercise for 8 weeks was able to improve renal SOD activity and reduce renal MDA [11].

In our findings, even with obesity, we found no evidence of renal inflammation or increased oxidative stress in the MetS group. Still, ET performed over 6 weeks was beneficial in increasing levels of anti-inflammatory interleukin (IL-10), being able to improve the anti-inflammatory profile of animals with MetS. However, the two groups that performed ET had reduced TAS and nitrite levels as compared to the other groups. We found no statistical difference in TOS and TBARS.

In the kidney, it is known the involvement of NO in the autoregulation and modulation of tubular transport, being of great importance in the development and progression of some kidney diseases (CKD or DKD; chronic kidney disease and diabetes kidney disease, respectively) [54]. We did not observe the statistically significant reduction of nitrite in the kidneys of animals that consumed okra, however, when we performed the effect size analysis according to Cohen [55] we observed large effect size between the MetS and O groups (d = 1.313) and small effect size between MetS + O and MetS + ET (d = 0.422) thus suggesting that the association ET + O is more beneficial in reducing nitrite levels.

The antioxidant system protects normal renal tissue functions [38]. We know that physical exercise is more efficient in the enzyme system, i.e., SOD, catalase and GPx. GPx can decrease lipid peroxidation induced by reactive oxygen species, protecting membrane integrity. SOD transforms harmful superoxide radicals into hydrogen peroxide, which is decomposed into oxygen and water by GPx and catalase [38]. In addition, studies with diabetic rats have shown that O fractions were able to reduce lipid peroxidation [13, 56] and restore renal GPx, SOD and catalase levels [56]. Therefore, we chose to analyze this system as well and observed less GPx activation in the trained groups, signaling worse renal antioxidant activity. However, ET combined with O improved renal catalase activity compared to MetS group. SOD and catalase activities appear to be reduced in obese animals [8] and, in addition, these animals have a higher level of renal oxidative stress [35]. However, in the present study, SOD activity was not altered in animals with MetS and therefore it was not possible to observe the benefits of the interventions.

Antioxidant systems act synergistically to remove excess free radicals in the body and maintain homeostasis. When this system is unbalanced, oxidative stress results in tissue damage [38]. A study [57] with diabetic rats supplemented with O for 45 consecutive days demonstrated the strong antioxidant action of O, which could protect these animals from oxidative stress and, consequently, from kidney damage. In our study, animals with MetS did not develop inflammatory but we observed an interesting result in nitrite levels. The increase in anti-inflammatory cytokine may have contributed to a lower nitrite level and consequently lower lesion in the renal tissue of these animals. However, it is worth mentioning that our animals did not develop hyperglycemia and this effect also seems to be related to its hypoglycemic action, which may also explain the lack of antioxidant action of O used in isolation observed in our study.

Based on our findings, animals with MetS showed few renal changes and even so the interventions brought benefits, but further investigations need to be carried out in order to clarify whether MetS or any of the risk factors are the trigger of these changes. Many of the data found in the literature show that kidney damage is due to hyperglycemia and obesity. However, our animals did not develop hyperglycemia, and this may justify different results. Inflammation is described in the literature as a trigger for oxidative stress, which is also responsible for kidney changes. As we did not observe an increase in these two factors, we suggest that the renal damage found may be the result of renal overload through obesity, IR and elevated triglycerides. The data found of nitrite should be explored and we suggest that the lower kidney injury found in the group that associated exercise and okra resulted from the lower nitrite value in the renal tissue of these animals.

Studies with MetS and CKD are still scanty in the literature, and this made it difficult to compare our data, since they were mostly corroborated by studies of obesity associated or not with DM. Many studies that associate MetS with kidney disease use diets high in fructose and/or sucrose. In our study, the animals consumed a standard diet, which may justify a lower inflammatory profile and kidney injury.

## Conclusion

In conclusion, our animals with MetS had a worse degree of renal injury and fibrotic deposition in the renal tissue, even though they did not develop inflammatory, oxidative stress and renal function changes. All interventions were beneficial in reducing fibrosis, but only ET combined with O was able to improve the degree of renal tissue impairment. ET improved the anti-inflammatory state and nitrite, but the combination of ET with O was more beneficial as regards catalase activity. The consumption of O alone did not promote changes in inflammatory cytokines and oxidative stress in the kidney. In conclusion, ET combined or not with O seems to be beneficial in preventing the progression of renal disease when renal function is not yet altered.

## Limitations

Our animals, despite developing MetS, did not develop inflammation and oxidative stress imbalance as described in the literature. Therefore, it was not possible to observe the benefits of interventions on these factors. The time of diagnosis of MetS may not have been sufficient to develop these changes.
